# American Dental Association

**Published:** 1896-09

**Authors:** 


### EDITORIAL.

### American Dental Association.

The annual meeting of this body was held at Saratoga, N.Y., on the first Tuesday of August, 1896. There was about the usual number in attendance, though many familiar faces were absent, but a goodly number was present that would have been absent had the meeting been held in the West or South. When the meeting is held three or four times in succession in the far East, it becomes somewhat monotonous, still it may be all for the best, and come to think of it, we rather think it is.

There was much good work done at Saratoga, some most excellent papers were read, the discussions were good, but not as extensive as they have been at some other times. A most excellent spirit prevailed throughout. There was nothing to mar the harmony and social spirit of the occasion.

The American Dental Association is doing a vast amount of good, notwithstanding the criticisms that have so freely been indulged at times. It is the representative body of the profession of this country. It has been the agency that has set the pace for the progress of dentistry; no other dental organization in this country is so free from trammel. It is entirely free to do what ought to be done; it has done more to encourage and foster the formation of State and local societies than any other influence; it has had its hand on the educational system of the dental profession, and in such a way as to stimulate marked advance, and this, too, by such means as to avoid friction or jealousies. While the educational work has been, in detail, carried on by those especially engaged in that work, it has all the while felt the guiding hand of the American Dental Association. Legislation is a subject that has often been considered by this central body, and in such a way as to have a salutary influence. While the carrying out of the provisions of the various State laws regulating the practice of dentistry is in the hands of the various State Examining Boards, together with the National Board of Dental Examiners, the consideration of the subject at large is in the hands of this representative body. This reference to these lines of work indicates most clearly the importance and value of the American Dental Association as a guiding influence for the dental profession. But this is not all; its ability to present and give direction to scientific work can not be estimated, such has been and is being done in this direction, but more will be done in the future. Now, in view of these things it is proper to ask: Ought not the American Dental Association to have a far larger support by the profession at large than it has as yet received? Let every one answer this question for himself, and then promptly act upon his convictions in the matter; then let there be a meeting at Old Point Comfort next year in numbers and earnestness commensurate with the need and importance of the occasion. Let every body come to the wedding, having on the wedding-garment and his lamp filled and well trimmed.

